# Non-negative low-rank representation based on dictionary learning for single-cell RNA-sequencing data analysis

**DOI:** 10.1186/s12864-022-09027-0

**Published:** 2022-12-23

**Authors:** Juan Wang, Nana Zhang, Shasha Yuan, Junliang Shang, Lingyun Dai, Feng Li, Jinxing Liu

**Affiliations:** School of Computer Science, Qufu Normal University, Rizhao, China

**Keywords:** Dictionary learning, Low-rank representation, scRNA-seq data analysis, Subspace clustering, Cell type identification

## Abstract

In the analysis of single-cell RNA-sequencing (scRNA-seq) data, how to effectively and accurately identify cell clusters from a large number of cell mixtures is still a challenge. Low-rank representation (LRR) method has achieved excellent results in subspace clustering. But in previous studies, most LRR-based methods usually choose the original data matrix as the dictionary. In addition, the methods based on LRR usually use spectral clustering algorithm to complete cell clustering. Therefore, there is a matching problem between the spectral clustering method and the affinity matrix, which is difficult to ensure the optimal effect of clustering. Considering the above two points, we propose the DLNLRR method to better identify the cell type. First, DLNLRR can update the dictionary during the optimization process instead of using the predefined fixed dictionary, so it can realize dictionary learning and LRR learning at the same time. Second, DLNLRR can realize subspace clustering without relying on spectral clustering algorithm, that is, we can perform clustering directly based on the low-rank matrix. Finally, we carry out a large number of experiments on real single-cell datasets and experimental results show that DLNLRR is superior to other scRNA-seq data analysis algorithms in cell type identification.

## Introduction

The single-cell RNA-sequencing (scRNA-seq) technology is now a powerful tool that demonstrates unprecedented precision in exploring biological processes and disease mechanisms [1–3]. The scRNA-seq technology helps to reveal the heterogeneity and diversity between cells. In addition, it can discover new subtypes and rare cell species by effectively dissecting complex and heterogeneous cell clusters [4–6]. Analyzing scRNA-seq data can help researchers better understand complex biological problems. In the scRNA-seq data analysis, one of the relatively significant research is unsupervised cluster analysis, which aims to identify cell types by clustering cells using clustering algorithms [7]. The researchers have previously introduced several traditional clustering methods to analyze these single-cell data. For example, Hartigan et al. proposed the K-means [8], which is based on Euclidean distance to minimize the distance between cells in the same class. Later, Luxburg et al. proposed the famous spectral clustering (SC) algorithm [9]. Elhamifar et al. developed the SC method based on the sparse representation sparse subspace clustering (SSC), which further improved the sparsity of subspace clustering and effectively processed the noise in data [10]. Compared with the bulk RNA-seq data and microarray data, the major problem of clustering scRNA-seq data is that missing values often appear in scRNA-seq data. Because of the limitation of current technology, scRNA-seq some-times fails to capture the expression of genes, thus resulting in dropout events in the data. These dropout events cause data loss in the gene expression matrix. These problems may reduce the accuracy of the above traditional clustering methods for identifying cell subtypes on scRNA-seq data.

In recent years, a number of specific methods have been proposed to overcome the challenges posed by the inherent nature of scRNA-seq data. Xu and Su proposed a quasi-clique-based clustering algorithm called SNN-Cliq [11], which constructs a distance matrix based on the concept of shared nearest-neighbor (SNN) to represent the similarity between cells. Wang et al. proposed a similarity learning framework, SIMLR, which uses multi-kernel similarity learning to analyze the scRNA-seq data [12]. SIMLR is essentially a spectral clustering method, which learns an appropriate distance matrix from the data for dimension reduction, clustering and visualization. SC3 [13] combines multiple sub-clustering results to construct the consistency matrix. Park et al. proposed an improved multi-kernel spectral clustering method named MPSSC [14]. In the MPSSC method, they modified the spectral clustering framework by imposing sparse structure on the target matrix. Inspired by previous methods that use neighborhood information to measure cell-to-cell similarity, Jiang et al. proposed a new cell similarity measure called Corr [15]. Corr considers the expression patterns of surrounding cells from a global perspective based on the correlation of cell-pair differentiability. Zheng et al. proposed SinNLRR to learn more accurate similarity matrix by low-rank representation (LRR) model with nonnegative constraint [16]. SinNLRR can reduce the influence of noise on similarity and effectively obtain accurate and robust clustering results. However, most clustering methods divide subspace clustering into two steps: first, learning an affinity matrix that encodes the subspace memberships of samples; then, the clustering algorithm such as Normalized cutting (NCuts) [9] is used to obtain the final clustering result based on the learned affinity matrix. Because the clustering method used to obtain the clustering results is not necessarily suitable for the learned affinity matrix. Thus, these methods are not guaranteed to obtain the optimal clustering results. In addition, although LRR-based methods have achieved good results in scRNA-seq data analysis, they usually choose the original data as the dictionary. Due to the large number of missing values and high noise of scRNA-seq data, using the original data as a dictionary to represent the low dimensional subspace is not conducive to obtaining accurate LRR matrix.

Xu et al. proposed Concept Factorization (CF), which attempts to find the data representation by using the linear combination of original data points to represent the cluster center [17]. Inspired by the idea of CF, we reformulated the dictionary in the LRR model with a linear combination of the original data and propose a new LRR-based method called Non-negative Low-rank Representation based on Dictionary Learning (DLNLRR). In this method, instead of using a fixed dictionary, the dictionary is modeled as the linear combination of the original data. In the optimization process, with the update of linear combination coefficient, the dictionary will be updated accordingly. Therefore, DLNLRR can realize dictionary learning and acquisition of LRR at the same time. Importantly, updating the dictionary can reduce the impact of data noise on the mapping benchmark, which will help to accurately extract the low-dimensional subspace structure of high-dimensional data. Secondly, we try to accurately find the corresponding subspaces of high-dimensional data through factor decomposition. Specifically, we determine the number of clusters by reasonably selecting the dimension of dictionary matrix. If the number of learned subspaces is the same as the actual number of clusters, we can directly gather the sample points into the corresponding subspace according to the projection of high-dimensional data on the low-dimensional subspace. In other words, the clustering results can be obtained without the help of the spectral clustering algorithm. Unlike previous single-cell cluster analysis methods that use NCuts to obtain clustering results, DLNLRR can avoid the influence of clustering algorithms on the final results. In addition, we add the manifold graph regularization to DLNLRR. When high-dimensional spatial data are mapped to low-dimensional space, manifold graph regularization can preserve the local geometric structure of high-dimensional data, so as to ensure the smoothness of manifold structure embedded in the high-dimensional data. Finally, to validate the effectiveness of the proposed method, we carry out a large number of experiments on real scRNA-seq datasets. Through comparative experiments, it is found that DLNLRR has higher clustering ability than the state-of-the-art scRNA-seq data clustering algorithms.

## Related work

Before introducing the proposed model, we will review some related methods in this section, including LRR, manifold graph regularization and CF.

### Low-rank representation

Because of the good ability to grasp the global data structure and explore the low-dimensional subspace structure, LRR has attracted the attention of a large number researchers and achieved good results in the application of bioinformatics [18]. In the LRR method, each sample can be represented as a linear combination of the bases in a given dictionary, and LRR seeks the lowest rank representation in a given data sample set [19]. So, LRR can realize the low-dimensional representation of high-dimensional data and reduce the difficulty of high-dimensional data processing. Given the observed data matrix $X \in R^{m \times n}$ is a combination of unknown independent subspaces $S = \left[ S_{1} ,S_{2,} ...,S_{b}\right]$. The LRR is formulated as the following rank minimization problem:

1 $$\begin{aligned} {\underset{H,E}{\min}}\ rank(H) + \lambda \left\| E \right\| _{l} ,s.t.X = AH + E. \end{aligned}$$

Here, $\lambda$ is a parameter and *A* is a basis matrix, called a dictionary. *H* is the LRR matrix with respect to the dictionary *A*, and the column vector ${h_j}$ denotes the mapping of sample point *j* in the subspace. The matrix *E* denotes the noise in the original data. $\left\| \cdot \right\| _{l}$ indicates a certain regularization strategy. Because of the discreteness of the rank operator, the above optimization problem (1) is difficult to solve. Previous studies [20] proposed the convex relaxation form of the optimization problem:

2 $$\begin{aligned} {\underset{H,E}{\min}}\, \left\| H \right\| _{*} + \lambda \left\| E \right\| _{l} ,s.t.X = AH + E, \end{aligned}$$

where $\left\| \cdot \right\| _{*}$ represents the nuclear norm, which is the sum of all singular values of a matrix. Obviously, an appropriate the dictionary *A* enables the LRR matrix *H* to reveal the true subspace structure of the data.

### Graph regularization based on manifold

In high-dimensional data processing, graph regularization constraint based on manifold learning offers a practicable choice for capturing the local geometry in data. The regularization of graph is based on local invariance assumption that if two data points $x_{i}$, $x_{j}$ are close in the original data geometric distribution, then their mappings $h_{i}$ and $h_{j}$ in the new space also remains close [21, 22]. Therefore, the graph regularization can reveal the underlying local manifold structure in the original data. For the sake of restoring the local geometric relationship between data points, a simple method is to construct a connected graph to approximate the manifold. We use data points in matrix *X* as vertices of the connected graph. Then, the symmetric weight matrix *S* is defined, ${s_{ij}}$ is the weight of the edge that connects the vertex $x_{i}$ to the vertex ${x_j}$. In this paper, Gaussian kernel is used to construct symmetric weight matrix *S* as follows:

3 $$\begin{aligned} {s_{ij}} = \left\{ \begin{array}{c} e^{ - \frac{{{{\left\| {{x_i} - {x_j}} \right\| }^2}}}{{2{t^2}}}}\\ 0 \end{array}\right. \left. \begin{array}{c} \quad {{x_i} \in {N_k}\left( {{x_j}} \right) or\;{x_j} \in {N_k}\left( {{x_i}} \right) }\\ { otherwise} \end{array}, \right. \end{aligned}$$

where ${N_k}\left( x_{i} \right)$ denotes the set of *k* nearest neighbors of $x_{i}$. $\left\| {{x_i} - {x_j}} \right\|$ is the Euclidean distance between $\mathop x\nolimits _i$ and ${x_j}$, and *t* controls the width of the neighborhoods, which is 1 by default. Based on the local invariance assumption, the definition of graph regularization in low dimensional space is as follows:

4 $$\begin{aligned} \begin{array}{l} {\underset{H}{min}} \sum \limits _{i,j} {{s_{ij}}} {\left\| {{h_i} - {h_j}} \right\| ^2}\\ \mathrm{{ = }}{\underset{H}{\min}}\ \mathrm{{tr}}\left( {H\left( {D - S} \right) {H^T}} \right) \\ \mathrm{{ = }}{\underset{H}{\min}}\ \mathrm{{tr}}\left( {HL{H^T}} \right) \end{array} \end{aligned}$$

Here, $h_{i}$ and $h_{j}$ denote the mappings of $x_{i}$ and $x_{j}$ under some transformation [23]. *D* is a diagonal matrix, $d_{ii} = \sum \limits _{i,j} {{s_{ij}}}$ is its diagonal element. It is obvious that $d_{ii}$ is the sum of similarities of data point $x_{i}$. *L* is the graph Laplacian matrix [24].

### Concept factorization

Concept Factorization (CF) was first proposed by Xu et al. [17], which has attracted great attention in dimensionality reduction and data clustering. CF is a variation of Nonnegative Matrix Factorization (NMF) [25]. The goal of NMF is to decompose the data matrix $X \in {R^{m \times n}}$ into two matrix factors $U \in {R^{m \times k}}$ and $V \in {R^{n \times k}}$, so that $U{V^T}$ can provide a good approximation to *X*.

5 $$\begin{aligned} X \approx U{V^T} \end{aligned}$$

Each column of *U* can be regarded as the basis vector, and each column of ${V^T}$ is the *k*-dimensional representation of the original inputs relative to the new basis. NMF mainly analyze the data matrices whose elements are nonnegative. And NMF imposes the nonnegative constraint on *U* and ${V^T}$. In the CF model, each base ${u_j}$ is represented by a linear combination of data points.

6 $$\begin{aligned} u_{j} = \sum \limits _i {w_{ij}{\textrm{x}}_i } , \end{aligned}$$

where ${w_{ij}} \ge 0$. Let $W = \left[ {{w_{ij}}} \right] \in {R^{n \times k}}$. The idea of CF is to represent each concept (base) as a linear combination of all data points, and approximate each data point by a linear combination of these concepts. That is, given a data matrix *X*, the goal of CF is to find two nonnegative coefficient matrices $W \in {R^{n \times k}}$ and $V \in {R^{n \times k}}$. The coefficient matrices meet the following condition:

7 $$\begin{aligned} X \approx XW{V^T} \end{aligned}$$

Equation (7) actually factorizes the data matrix *X* into *X*, *W*, and *V*. *W* is referred to as the association matrix recording the concepts, while *V* represents the projection corresponding to the concept and is referred to as the representation matrix.

## Materials and methods

In this section, we will introduce the proposed DLNLRR method in detail, which uses the linear combination of original data as a dictionary to seek the lowest rank representation of data points.

### DLNLRR method

Given a high-dimensional data $X \in {R^{m \times n}}$ with *n* data points, the main goal of LRR is to find the lowest rank representation of data points based on a given dictionary. LRR-based method usually directly uses the original data matrix *X* as the dictionary to grasp the similarity matrix or LRR. However, scRNA-seq data usually contain a lot of noise and missing values. In view of the characteristics of scRNA-seq data, directly using the original data as the dictionary can not accurately represent the basis of low-dimensional projection. Inspired by the idea that CF represents concepts through a linear combination of all data points, we attempt to model the dictionary *A* as a linear combination of original data points, i.e., $A = XW$. We can update and reconstruct the dictionary by updating the coefficient matrix *W*, so that the dictionary can better represent the low dimensional subspace through continuous learning. Compared with using the original data as a dictionary, the dictionary with learning ability is helpful to further identify the potential low dimensional subspace structure. In addition, to improve the interpretability of the model, we impose the non-negative constraint on *H*. So, we have

8 $$\begin{aligned} \begin{aligned} {\underset{W,H}{\min}}\ \left\| H \right\| _{*} ,s.t.X = XWH,W{W^T} = I,H \ge 0, \end{aligned} \end{aligned}$$

where $W \in R^{n \times r}$ is the coefficient matrix, the orthogonal constraint $W{W^T} = I$ is to ensure that the model is stable. *r* denotes the number of subspaces. *XW* is referred to as concept matrix in the CF model. If we regard a concept as a subspace, *XW* can be regarded as the dictionary *A* in the LRR model. Therefore, the dictionary *A* can be continuously updated with the iteration of *W* during the optimization process. $H \in {R^{r \times n}}$ is the LRR of the original data *X* with respect to dictionary *A*. Each column of *H* represents the mapping of a sample point in a low dimensional subspace. So *H* can intuitively reflect the non-negative similarity of different type of cells. When the data is projected from high-dimensional space to low-dimensional space, manifold graph regularization can maintain the inherent local geometry of the data. Therefore, we introduce the regularization of manifold graph into problem (8) to constrain the low-rank matrix *H*. The introduction of manifold graph regularization can ensure the smoothness of the nonlinear manifold structure embedded in the high-dimensional data. In addition, we relax the constraint $X = XWH$ to minimize $X - XWH$. The mathematical model of our method is as follows:

9 $$\begin{aligned} {\underset{W,H}{\min}}\ \frac{1}{2}{\left\| {X - XWH} \right\| }_{F}^{2} + \lambda {\left\| H \right\| }_{*} + \beta \mathrm{{tr}}\left( {HL{H^T}} \right) ,s.t.W{W^T} = I,H \ge 0. \end{aligned}$$

Here, ${\left\| \cdot \right\| }_{F}$ denotes the Frobenius norm, which is the square root of the sum of squares of all elements in a matrix. $\lambda$, $\beta$ are the penalty parameters. We refer to model (9) as Non-negative Low-rank Representation based on Dictionary Learning (DLNLRR). By optimally solving the DLNLRR model, we can obtain an LRR matrix *H* with *r* rows and *n* columns. We expect the LRR matrix $H \in {R^{r \times n}}$ to have a clear clustering structure, i.e., the *r* low-dimensional subspaces found correspond to *r* clusters. Thus, the element $h_{ij}$ represents the projection of sample *j* on subspace *i*, and we can directly cluster the samples based on the maximum of each column vector in the LRR matrix. For example, if $h_{ij}$ is the maximum value in the *j*-th column, sample *j* will be clustered into cluster *i*. So, we can obtain the clustering labels directly without resorting to other clustering algorithms, which avoids the influence of clustering methods on the clustering results and then improves the clustering performance of our method. Through the above analysis, we can see that the correct selection of the value of *r* is the key to the direct clustering of sample points. In Section ‘Selection of Dimension *r*’, we will discuss how to select the appropriate *r* value.

### Optimization

The objective function of DLNLRR is a convex optimization problem with multiple constraints. In this subsection, we use the penalty term adaptive linear alternating direction (LADMAP) method [26] to settle the matter defined by problem (9). Firstly, we introduce two auxiliary variables *J* and *Z*, problem (9) is rewritten as

10 $$\begin{aligned} {\underset{J,Z,W,H}{\min}}\ \frac{1}{2}{\left\| {X - XZ} \right\| }_{F}^{2} + \lambda {\left\| J \right\| }_{*} + \beta \mathrm{{tr}}\left( {HL{H^T}} \right) , s.t.Z = WH,J = H,W{W^T} = I,H \ge 0. \end{aligned}$$

Secondly, we introduce the augmented Lagrangian function to eliminate the linear constraints in problem (10). Therefore, problem (10) can be expressed as following:

11 $$\begin{aligned}{} & {} L\left( {J,Z,W,H,{Y_1},{Y_2}} \right) = \frac{1}{2}{\left\| {X - XZ} \right\| }_{F}^{2} + \lambda {\left\| J \right\| }_{*} + \beta \mathrm{{tr}}\left( {HL{H^T}} \right) \nonumber \\ +{} & {} \left\langle {{Y_1},Z - WH} \right\rangle + \left\langle {{Y_2},J - H} \right\rangle + \frac{\mu }{2}{\left\| {Z - WH} \right\| }_{F}^{2} + \frac{\mu }{2}{\left\| {J - H} \right\| }_{F}^{2} , \end{aligned}$$

where $\mu$ is a penalty parameter, ${Y_1}$ and ${Y_2}$, are the Lagrange multipliers. Finally, we update *J*, *Z*, *W* and *H* sequentially using the alternating minimization strategy, that is, when you update one variable, keep the other variables unchanged.

####  Updating J

To update the variable *J*, according to problem (11), the subproblem with respect to *J* is converted to

12 $$\begin{aligned} \begin{array}{l} {\underset{J}{\min}}\ \lambda {\left\| J \right\| }_{*} + \left\langle {{Y_2},J - H} \right\rangle + \frac{\mu }{2}{\left\| {J - H} \right\| }_{F}^{2} \\ = {\underset{J}{\min}}\ \lambda {\left\| J \right\| }_{*} + \frac{\mu }{2}\left\| {J - H + \frac{{{Y_2}}}{\mu }} \right\| _{F}^{2}. \end{array} \end{aligned}$$

It can be solved by soft-thresholding.

13 $$\begin{aligned} J \leftarrow sof{t_{\lambda ,1/\mu }}\left( {H - \frac{{{Y_2}}}{\mu }} \right) , \end{aligned}$$

where $sof{t_{\lambda ,1/\mu }}\left( \cdot \right)$ represents the soft-thresholding operator [19]. And $sof{t_{\lambda ,1/\mu }}\left( X \right) = U{D_{\lambda ,1/\mu }}\left( \Sigma \right) {V^T}$, $X = U\Sigma {V^T}$. The element on the diagonal of matrix $\Sigma$ is ${\sigma _{ii}}$, ${D_{\lambda ,1/\mu }}\left( \Sigma \right) = diag\left( {\max \left( {\sigma _{ii} - \lambda / \mu ,0} \right) } \right)$.

#### Updating Z

To update the variable *Z*, according to problem (11), the subproblem with respect to *Z* is as follows.

14 $$\begin{aligned} {\underset{Z}{\min}}\ \frac{1}{2}{\left\| {X - XZ} \right\| }_{F}^{2} + \left\langle {{Y_1},Z - WH} \right\rangle + \frac{\mu }{2}{\left\| {Z - WH} \right\| }_{F}^{2}\nonumber \\ = {\underset{Z}{\min}}\ \frac{1}{2}{\left\| {X - XZ} \right\| }_{F}^{2} + \frac{\mu }{2}\left\| {Z - WH + \frac{{{Y_1}}}{\mu }} \right\| _{F}^{2}. \end{aligned}$$

We differentiate our objective function with respect to *Z*, and then we set it to zero. We get

15 $$\begin{aligned} Z = {\left( {2{X^T}X + \mu I} \right) ^{ - 1}}\left( {2{X^T}X + \mu WH - {Y_1}} \right) . \end{aligned}$$

####  Updating W

Similarly, the subproblem with respect to *W* is as follows.

16 $$\begin{aligned} {\underset{W}{\min}}\ \left\langle {{Y_1},Z - WH} \right\rangle + \frac{\mu }{2}{\left\| {Z - WH} \right\| }_{F}^{2} ,\ s.t.{W^T}W = I. \end{aligned}$$

Problem (16) can be rewritten as

17 $$\begin{aligned} {\underset{W}{\min}}\ \frac{\mu }{2}\left\| {WH - \left( {Z + \frac{{{Y_1}}}{\mu }} \right) } \right\| _{F}^{2},\ s.t.{W^T}W = I. \end{aligned}$$

In order to solve *W*, let $Q = Z + \frac{{{Y_1}}}{\mu }$, so the objective function of (17) is equivalent to the following formula:

18 $$\begin{aligned} {\underset{W}{\min}}\ \frac{\mu }{2}{\left\| {WH - Q} \right\| }_{F}^{2} ,\ s.t.{W^T}W = I. \end{aligned}$$

Derivation of formula (18):

19 $$\begin{aligned} \begin{array}{l} {\underset{W}{\min}}\ {\left\| {WH - Q} \right\| }_{F}^{2}\\ = {\underset{W}{\min}}\ Tr\left( {{H^T}{W^T}WH} \right) - 2Tr\left( {WHQ^{T} } \right) + Tr\left( {Q^{T} Q} \right) \\ = {\underset{W}{\min}}\ Tr\left( {WHQ^{T}} \right) ,\ s.t.{W^T}W = I. \end{array} \end{aligned}$$

For (19), the Lagrangian function *L* is constructed using symmetric matrix multipliers of $\Lambda$.

20 $$\begin{aligned} L\left( {W,\Lambda } \right) = Tr\left( {WHQ^{T}} \right) - Tr\left( {{\Lambda ^T}\left( {{W^T}W - I} \right) } \right) /2. \end{aligned}$$

Then,

21 $$\begin{aligned} L_{w} = {QH}^{T} - W\Lambda = 0,\mathrm{{ }}i.e.\mathrm{{ }}\Lambda = {W^T}QH. \end{aligned}$$

Thus, ${\Lambda ^T}\Lambda = {\Lambda ^T}{W^T}W\Lambda = HQ^{T} Q{H^T} = V\Omega {U^T}U\Omega {V^T}$, since $\Lambda = {\Lambda ^T}$, $\Lambda = V\Omega {V^T}$. From (21), the optimal *W* is given by the singular vectors:

22 $$\begin{aligned} W = U{V^T},Q{H^T} = U\Omega {V^T},\Omega = diag\left( \omega \right) , \end{aligned}$$

where $\left( {U,\Omega ,V} \right)$ is the SVD decomposition of $Q{H^T}$.

####  Updating H

We update *H* by solving the following problems

23 $$\begin{aligned} {\underset{H}{\min}}\ \beta \mathrm{{tr}}\left( {HL{H^T}} \right) + \left\langle {{Y_1},Z - WH} \right\rangle + \left\langle {{Y_1},J - H} \right\rangle {} + \frac{\mu }{2}{\left\| {Z - WH} \right\| }_{F}^{2} + \frac{\mu }{2}{\left\| {J - H} \right\| }_{F}^{2}\nonumber \\ = {\underset{H}{\min}}\ \beta \mathrm{{tr}}\left( {HL{H^T}} \right) + \frac{\mu }{2}\left\| {Z - WH + \frac{{{Y_1}}}{\mu }} \right\| _{F}^{2} + \frac{\mu }{2}\left\| {J - H + \frac{{{Y_2}}}{\mu }} \right\| _{F}^{2}. \end{aligned}$$

By taking the derivative of the function with respect to *H* and setting it to zero, we find that the optimal solution *H* should satisfy

24 $$\begin{aligned} \mu \left( {{W^T}W + I} \right) H + H\left( {2\beta L} \right) + \left( {{Y_2} - \mu W^{T} Z - W^{T} Y_{1} - \mu J} \right) = 0. \end{aligned}$$

Problem (24) is looks like

25 $$\begin{aligned} AX + XB + Q = 0, \end{aligned}$$

where $A \in R^{m \times m}$, $B \in R^{n \times n}$ and $Q \in R^{m \times n}$ are three given matrices, and *X* is a matrix to be solved. This is a standard Sylvester equation that has a unique solution for *X* if and only if no eigenvalue of *A* is the negative of an eigenvalue of *B* [27]. Thus, we directly use the lyap function in matlab to solve for *H* according to problem (24). After all variables are updated, these Lagrange multipliers are updated by

26 $$\begin{aligned} {Y_1} = {Y_1} + \mu \left( {Z - WH} \right) , \end{aligned}$$

27 $$\begin{aligned} {Y_2} = {Y_2} + \mu \left( {J - H} \right) . \end{aligned}$$

The parameter $\mu$ is updated by $\mu = \min \left( {\rho \mu ,{\mu _{\max }}} \right)$, after all variables and multipliers have been updated [18]. The main procedure of DLNLRR is described in Algorithm 1.

**Figure:**
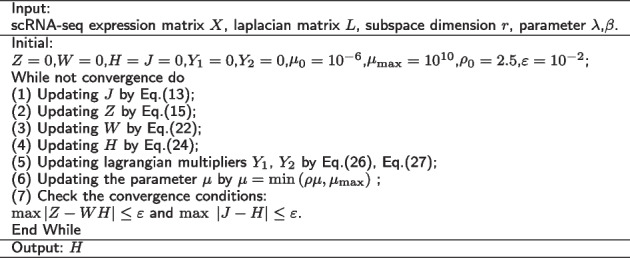
**Algorithm 1** The main procedure of DLNLRR.

### Framework for DLNLRR

**Fig. 1:**
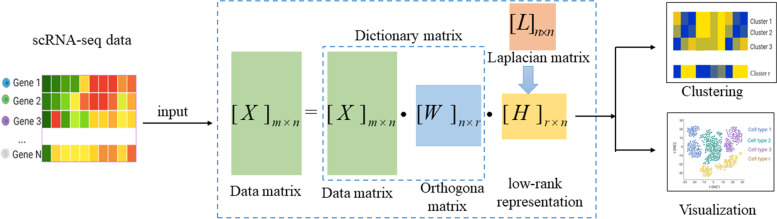
The DLNLRR algorithm framework

In this subsection, we will describe the framework of DLNLRR. DLNLRR consists of two basic steps, including low-rank matrix learning, downstream analysis. The framework of the DLNLRR algorithm is shown in Fig. 1. Given a scRNA-seq expression matrix, to reduce the technical noise in each scRNA-seq dataset, we first pre-process the data by gene filtering and median normalization. In the gene filtering, we remove bad genes expressed in less than or equal to two cells. In the median normalization, the raw read count is normalized by the size factor, followed by a log transformation ${\log }_{10} \left( {x + 1} \right)$. Then, the preprocessed data are input to the DLNLRR model. After continuous iterative solving, we obtain the LRR matrix *H* of the original data matrix relative to the dictionary. As mentioned earlier, if the appropriate *r* value is selected, we can cluster the samples according to the maximum value of each column vector in the LRR matrix *H* to obtain the final clustering result. Finally, the t-SNE algorithm is used to visualize the LRR matrix *H* to validate the effectiveness of DLNLRR in learning cell-to-cell similarity from scRNA-seq data.

### Running time and memory usage

The DLNLRR method runs on PC with i5-10210U CPU @ 1.60GHz and 16.0G RAM. We tested the running time of the algorithm developed by the MATLAB on eight single cell datasets with different cell numbers. The actual running time of the algorithm is all the running steps of each method, including data preprocessing. Table 1 shows the actual computation time. We find that the running time of most algorithms increases with the increase of the number of samples. DLNLRR and sinNLRR are both LRR based methods, which run faster on single cell data and take less than 2 minutes on all eight data sets. Since the sinNLRR method does not carry out dictionary learning, its running time is faster. However, from clustering experiments, it is found that our method can obtain more accurate results. In addition, MPSSC is a multiple kernel based method that requires more running memory.

**Table 1:** Running time

Methods	Treutlein	Ting	Deng	Pollen	Goolam	Engel4	Kolod	Darmanis
SSC	4.64s	6.79s	6.25s	25.34s	21.83s	42.23s	174.33s	93.44s
SNN-Cliq	12.33s	22.67s	27.59s	42.34s	39.79s	82.47s	192.66s	78.26s
Corr	11.51s	289.67s	358.01s	1931.77s	976.91s	1023.76s	5733.45s	3352.41s
MPSSC	4.72s	4.51s	5.69s	7.12s	4.59s	4.75s	31.28s	11.94s
SinNLRR	0.63s	0.83s	0.89s	1.77s	0.79s	1.61s	25.02s	35.97s
DLNLRR	1.03s	2.04s	2.64s	7.42s	3.62s	7.09s	77.98s	32.58s

## Results and discussion

### scRNA-seq datasets

We tested the DLNLRR method across eight scRNA-seq datasets generated by different platforms. We downloaded these data sets from databases provided by the National Biotechnology Information Retrieval Database (NCBI) and the European Institute for Bioinformatics (EMBL-EBI). Specifically, these datasets include Treutlein [28], Ting [29], Deng [30], Pollen [31], Goolam [32], Engel [33], Kolod [34], Darmanis [35]. The brief description of the eight scRNA-seq datasets is listed in Table 2. They are observed to vary in sample size from 80 (Treutlein) to 704 (Kolod), and the number of cell clusters ranges from 3 (Kolod) to 11 (Pollen).

**Table 2:** The scRNA-seq datasets

Datasets	Number of cells	Number of genes	Cell types	Species
Treutlein	80	959	5	Mus musculus
Ting	114	14405	5	Mus musculus
Deng	135	12548	7	Mus musculus
Pollen	249	14805	11	Homo sapiens
Goolam	124	40315	5	Mus musculus
Engel	203	23337	4	Homo sapiens
Kolod	704	10685	3	Mus musculus
Darmanis	420	22085	8	Homo sapiens

### Evaluation measurements

In this experiment, we used Normalized Mutual Information (NMI) [36] and Adjusted Rand Index (ARI) [37, 38] to validate the performance of the proposed method. Both NMI and ARI can be used to compare the agreement of data distribution between clustering algorithm and real clustering labels. NMI is an evaluation standard to detect the degree of difference between two types of clustering results according to the relationship between joint entropy and individual entropy. NMI measures the mutual information between the obtained clustering labels and the truth labels, followed by a normalization operation to assure NMI ranges from 0 to 1.

Let $M = \left\{ {{M_1},{M_2}, \cdot \cdot \cdot ,{M_K}} \right\}$ and $N = \left\{ {{N_1},{N_2}, \cdot \cdot \cdot ,{N_K}} \right\}$ represent the known real cluster and the inferred cluster by some clustering method respectively. Mathematically, NMI is defined as:

28 $$\begin{aligned} NMI\left( {M,N} \right) = \frac{{2MI\left( {M,N} \right) }}{{H\left( M \right) + H\left( N \right) }}, \end{aligned}$$

where $H\left( \cdot \right)$ represents the entropy of the cluster and $MI\left( { \cdot , \cdot } \right)$ represents the mutual information among cluster [39]. ARI is a kind of evaluation criterion to measure the consistency between real clusters *M* and inferred clusters *N*. Mathematically, it is defined as:

29 $$\begin{aligned} ARI\left( {M,N} \right) = \frac{{\left( {\begin{array}{l} n\\ 2 \end{array}} \right) \left( {a_{mn} + a} \right) - \left[ {\left( {a_{mn} + a_{m} } \right) \left( {a_{mn} + a_{n} } \right) + \left( {a_{n} + a} \right) \left( {a_{m} + a} \right) } \right] }}{{\left( {\begin{array}{l} n\\ 2 \end{array}} \right) - \left[ {\left( {a_{mn} + a_{m}} \right) \left( {a_{mn} + a_{n}} \right) + \left( {a_{n} + a} \right) \left( {a_{m} + a} \right) } \right] }},\ \end{aligned}$$

where$a_{mn}$ represents the number of a pair of objects placed in the same group in *M* and *N*, $a_{m}$ represents the number of pairs in the same group *M* but in the different groups in *N*, $a_{n}$ represents the number of pairs in the same group in *N* but in the different groups in *M*, *a* represents the number of objects in a pair that are placed in the different groups in *M* and *N*. The value range of NMI and ARI are [0,1]. In general, if the values of NMI and ARI are close to 1, it indicates that the clustering results are close to the real situation.

### Selection of Dimension r

**Fig. 2:**
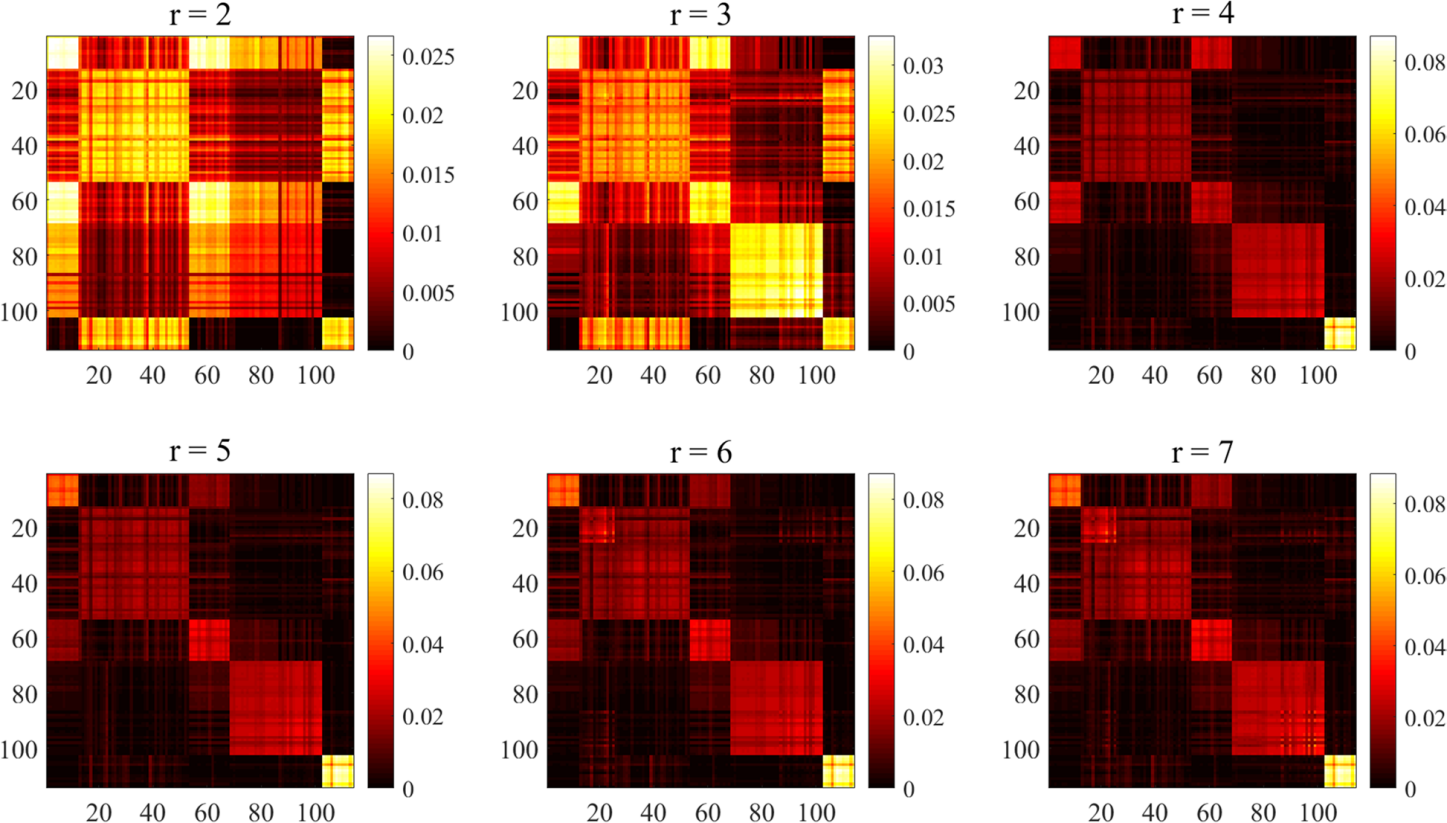
The heatmaps of $\left| {{H^T}H} \right|$ on Ting dataset

According to section ‘DLNLRR Method’, each column vector of the LRR matrix $H \in R^{r \times n}$ is a new representation of the original data set in the low-dimensional subspace. Each subspace found by DLNLRR corresponds to an actual cluster. Therefore, the selection of dimension *r* of LRR matrix *H* becomes the key of this method, which will affect the accuracy of clustering results. We choose the Ting dataset as an example to further verify the influence of different dimensions *r* on the learning low-rank matrix. To more intuitively demonstrate the learning ability of low rank matrix to subspace structure, we show the heatmap of $\left| {{H^T}H} \right|$ under different dimensions *r* in Fig. 2. In ideal state, $\left| {{H^T}H} \right|$ should have the clear block diagonal structure. The block structure presented by the heat map of $\left| {{H^T}H} \right|$ reflects the clustering structure, i.e., the clearer the block diagonal structure is, the more desirable the clustering result is. If *r* is chosen appropriately, the number of presented blocks should be consistent with the number of the cell types. It can be seen from Fig. 2 that when $r = 2$ and $r = 3$, $\left| {{H^T}H} \right|$ is divided into several blocks, but non-diagonal areas are also bright, even brighter than the diagonal area. This indicates that the value of *r* is not appropriate, and its value is lower than the real number of clusters, resulting in considerable similarity between different types of cells. Obviously, it is inappropriate to set $r = 2$ and $r = 3$. When $r = 4$, $\left| {{H^T}H} \right|$ has the clear block diagonal structure and is divided into five modules. However, the number of modules does not match the value of *r*. According to the previous discussion, subspace clustering can be realized directly based on *H* only when the value of *r* is consistent with the number of clustering clusters. We note that when $r = 5$, there is no significant difference compared with $r = 4$, and the number of blocks does not increase. When $r = 6$ and $r = 7$, the number of diagonal blocks is still 5. This indicates that the number of blocks does not increase with the increase of *r*. So, we can infer that the number of clusters is 5. In order to achieve direct clustering, we choose the *r* value consistent with the number of diagonal blocks, that is, $r = 5$. Therefore, in the Ting dataset, *r* is set to 5.

### Parameter selection

**Fig. 3:**
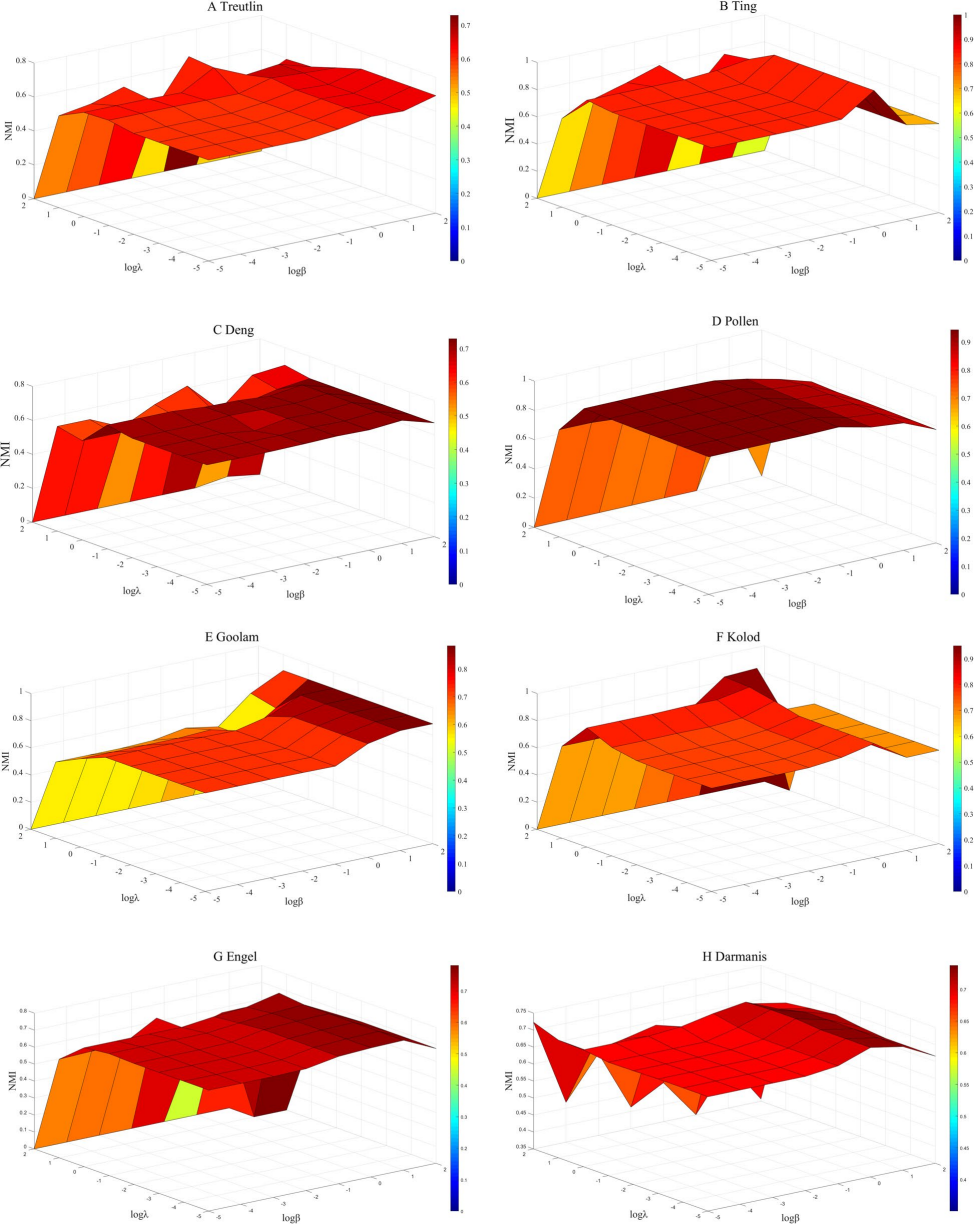
Influence of parameters $\lambda$ and $\beta$ on NMI

This subsection analyzes the impact of parameters in DLNLRR on the clustering performance. According to formula (9), there are two parameters $\lambda$ and $\beta$ in the DLNLRR model. We use grid search to find the most appropriate parameters on each dataset. We let the two parameters vary in the interval [$10^{-5}$, $10^2$] and show the NMI on eight datasets in Fig. 3. From Fig. 3, it is found that except Pollen dataset, the other seven datasets with $\lambda$ in [$10^{-1}$ $\sim$ $10^1$] and $\beta$ in [1 $\sim$ 10] can obtain satisfactory results. For Pollen dataset, we can obtain the best results when $\lambda \mathrm{{ = 1}}{\mathrm{{0}}^\mathrm{{0}}}$, ${} \beta = {10^{ - 3}}$. Then, through further search, we list the optimal parameters of each dataset in Table 3.

**Table 3:** The optimal parameters on each dataset

Datasets	parameter $\lambda$	parameter $\beta$
Treutlein	10$^{0.6}$	7
Ting	10$^{-1}$	1
Deng	10$^1$	7
Pollen	10$^0$	10$^{-3}$
Goolam	10$^0$	8
Engel	10$^{0.8}$	7
Kolod	10$^{0.8}$	6
Darmanis	10$^{0.8}$	7

### Clustering performance of DLNLRR

**Fig. 4:**
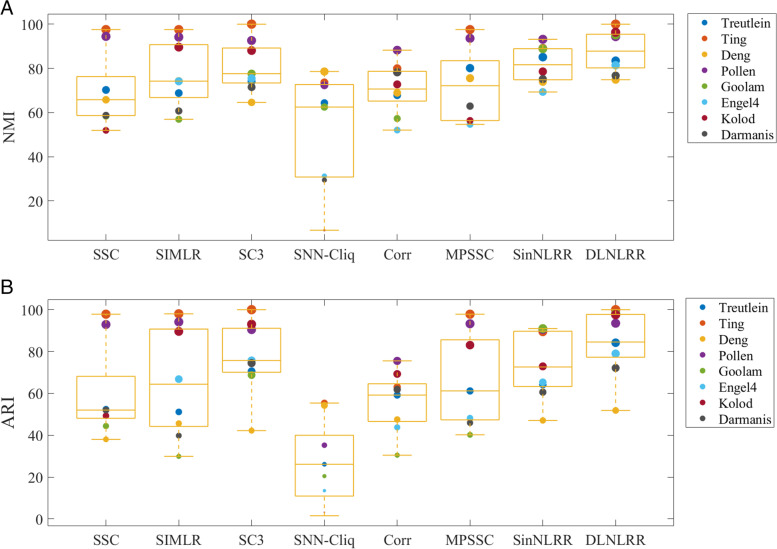
Various methods on eight datasets of NMI and ARI

Cell clustering is one of the important tasks in the mining and analysis of scRNA-seq data, and its main purpose is to distinguish cell types. In this subsection, to validate the clustering performance of DLNLRR, DLNLRR and several most advanced single-cell data clustering methods are applied to cluster cells on eight real scRNA-seq datasets described in Table 2. Specifically, the compared methods include SSC [10],SIMLR [12], SC3 [13], SNN-Cliq [11], Corr [15], MPSSC [14], SinNLRR [16], ScLCA [40], Seurat [41], CIDR [42], RaceID [43], Spectrum [44], and SHARP [45],respectively. The procedures for the above comparison methods can be found in the scRNA tool database [46, 47]. We used NMI and ARI introduced in Section ‘Evaluation measurements’ to evaluate the performance of the clustering method. Table 4 shows the specific ARI values of the above methods.To reflect the overall clustering performance of each clustering method on all data sets, we show the experimental results of SSC, SIMLR, SC3, SNN-Cliq, Corr, MPSSC, SinNLRR and DLNLRR in the form of Box-plot in Fig. 4.

**Table 4:** The percentage of specific ARI values of clustering methods

methods	Treutlein	Ting	Deng	Pollen	Goolam	Engel	Kolod	Darmanis	Average
SSC	52.42	97.84	38.04	92.92	44.41	52.02	49.30	52.02	59.87
SIMLR	51.14	98.03	45.65	94.15	29.91	66.82	89.60	39.82	64.39
SC3	70.56	100.00	42.21	90.45	68.74	75.70	93.06	74.46	76.89
SNN-Cliq	26.13	55.37	54.26	35.24	20.46	13.52	3.12	1.53	26.20
Corr	59.19	63.02	47.53	75.53	30.46	43.77	69.2	61.83	56.32
MPSSC	61.17	97.84	47.83	93.28	40.20	48.21	83.06	45.93	64.69
SinNLRR	64.19	89.43	47.06	90.51	90.97	65.33	72.91	60.57	72.62
ScLCA	71.57	44.95	36.27	87.29	49.14	26.38	83.40	81.76	60.09
Seurat	62.50	75.63	54.96	82.32	58.21	70.87	72.32	88.30	70.63
CIDR	93.29	-	43.01	81.39	-	18.52	-	89.77	65.19
RaceID	69.62	-	52.09	83.83	-	49.66	-	50.78	61.19
Spectrum	42.23	56.78	31.34	89.24	52.00	09.10	43.27	36.46	45.05
SHARP	71.39	82.08	64.42	42.12	93.87	69.99	84.42	94.88	75.39
DLNLRR	84.20	100.00	51.84	93.52	98.08	79.01	97.62	72.14	84.55

Firstly, it can be seen from Table 4 that although our method does not achieve the best results on all data sets, DLNLRR has achieved ARI values greater than 50$\%$ on all data sets and the highest average value, indicating its robustness. In addition, no other method can obtain the highest ARI value on more than two datasets.

Secondly,we can see from Fig. 4 that the position of the box generated by our method is relatively high compared with other methods. In addition, we can find that the median line of DLNLRR is the highest of all methods. This indicates that our method has the best overall performance on all datasets. And, the compactness of the box in Box-plot shows the stability of the performance of the method. As can be seen from Fig. 4A, the boxes for SC3, Corr, SinNLRR and DLNLRR are relatively compact. This shows that the performance of these four methods is relatively stable. Similar results can be found for ARI in Fig. 4B. Summarizing the above analysis, we can conclude that DLNLRR is stable and efficient in scRNA-seq data clustering. In addition, we further compare our method with SinNLRR. Because our method and SinNLRR are both based on LRR model. The main difference between the two methods is that the SinNLRR method directly uses the original data as a fixed dictionary, while DLNLRR uses the linear combination of the original data as the dictionary to update the dictionary in iterations. As can be seen from Fig. 4, our method is superior to SinNLRR in NMI and ARI value. Compared with the average performance of SinNLRR, the average NMI and ARI of our method are increased by 0.119 and 0.061, respectively. We can infer that updating the dictionary in the optimization process instead of using the predefined fixed dictionary can more accurately learn the structural information in the data and improve the clustering performance of single-cell data.

### Visualization and gene markers

#### Visualization analysis

**Fig. 5:**
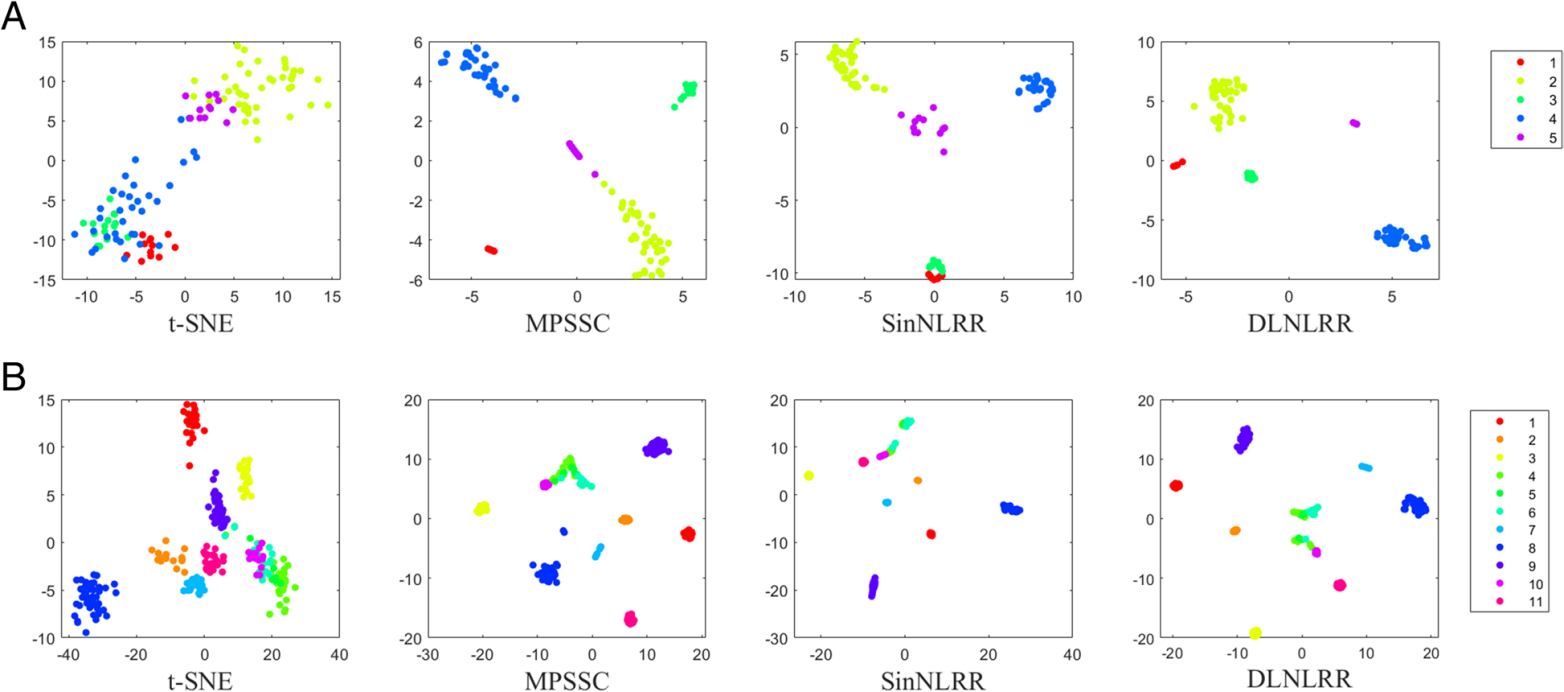
Visualization results for t-SNE, MPSSC, SinNLRR, and DLNLRR

Visualizing scRNA-seq data in low-dimensional space is a powerful way to pre-identify cell subpopulations. Previous studies proposed an improved t-distributed Stochastic Neighbor Embedding (t-SNE) for dimensionality reduction and visualization of data to verify the performance of the learned similarity matrix [48]. In this subsection, to investigate the performance of DLNLRR in learning intercellular similarity from original scRNA-seq data, we input the low-rank matrix learned by DLNLRR into t-SNE to visualize scRNA-seq data. We shown the visualization results of t-SNE, MPSSC, SinNLRR and DLNLRR on Ting dataset and Pollen dataset in Fig. 5. In Fig. 5, dots with the same color indicate that they have the same cell type. Among the four methods, t-SNE visualizes the data directly based on the original single-cell expression data, while for MPSSC, SinNLRR and DLNLRR method, the data is visualized based on the obtained similarity matrix. Therefore, we first compare t-SNE with MPSSC, SinNLRR and DLNLRR. As can be seen from Fig. 5, in the visualization of t-SNE, the cells of various cell types are mixed and cannot be well separated, whether on Ting or Pollen. Compared with t-SNE, the cells of different cell types can be clearly separated in the visualization of MPSSC, SinNLRR and DLNLRR. This indicates that the similarity matrix learned by MPSSC, SinNLRR and DLNLRR can better reflect the structural information of data points in low-dimensional subspace. Secondly, we compare DLNLRR with MPSSC and SinNLRR. From Fig. 5A, we can find that, compared with SinNLRR and MPSSC, the visualization of DLNLRR can better reflect the distribution law in the data. Specifically, in the visualization of DLNLRR on Ting dataset, the cells of the same type are highly aggregated, while the cells of different types are clearly distinguishable. This shows that the learning dictionary in DLNLRR is conducive to grasp the subspace structure of high-dimensional data. Finally, we would like to further explain the visualization of DLNLRR on Pollen dataset. As shown in Fig. 5B, for the Pollen dataset of 11 clusters, no method can completely separate the clusters. Compared with t-SNE and MPSSC, the DLNLRR method shows less overlap and compact-ness. Compared with SinNLRR, the DLNLRR method shows better distinguish ability between different types of data.

#### Gene markers

**Fig. 6:**
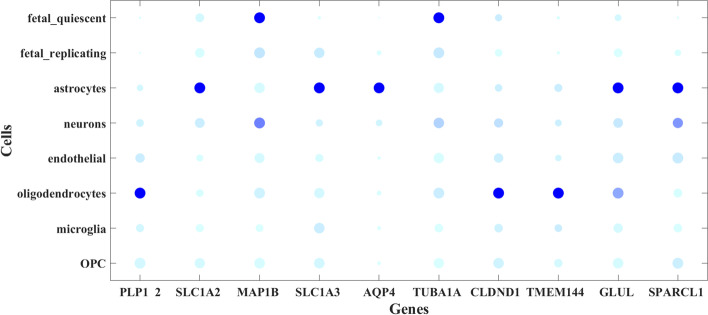
The top 10 gene markers in Darmanis datasets. Gene names are plotted on the X-axis and cell types on the Y-axis

Gene marker prioritization has attracted extensive attention since it was proposed. Gene markers have rich in biological information, which helps to distinguish cell subpopulations and reveals the complexity of cells. In this subsection, we identify the gene markers for each cell type in the Darmanis dataset based on the learned low-rank matrix. First, Bootstrap Laplacian scoring [12] is performed on the low-rank matrix to extract its gene markers. Then, the gene markers are arranged in de-scending order according to their importance in identify-ing subsets of cells. The top 10 gene markers in the Damanis dataset are shown in Fig. 6. In Fig. 6, we use the depth of color to express their expression level. The darker the color is, the higher the expression level is. The size of the circle indicates the percentage of genes expressed in each cell. The Darmanis dataset contains 420 brain cells from fetuses and adults. They included 16 microglia, 18 oligodendrocyte progenitor cells (OPC), 20 endothelial cells, 25 fetal replating neurons, 38 oligodendrocytes, 62 astrocytes, 110 fetal quiescent neurons, and 131 neurons [35]. In Fig. 6, the MAP1B, TUBA1A genes get trapped in the development of fetal quiescent neurons and become important components of cell survival and differentiation [49, 50]. Furthermore, MAP1B gene over-expression is also connected with neuronal activation. The SLC1A2, SLC1A3, AQP4, GLUL and SPARCL1 genes have been confirmed to be highly expressed in astrocytes, and their mutations or mutations are often closely related to various diseases [16, 51, 52]. PLP12, CLDND1 and TMEM144 genes are stably expressed in the myelin of oligodendrocytes [53]. The protein encoded by PLP12 may play important roles in myelin compaction, stabilization and maintenance, and promote oligodendrocyte development and axon survival. The protein encoded by AQP4 is the main aquaporin in the brain and plays a key role in cerebral hydro homeostasis. Articles published confirm PLP12 and AQP4 were astrocytes and oligodendrocytes marker genes [54].

## Conclusions

The development of scRNA-seq and high-throughput technologies has facilitated the exploration of single-cell function and brings computational challenges to reveal the relationship between cell lineages. Cell clustering and extraction of gene markers are important research components of analyzing scRNA-seq data. In this paper, we propose the DLNLRR method for scRNA-seq data analysis. Inspired by the idea of CF, DLNLRR uses the linear combination of original data to construct the dictionary. Instead of using the predefined dictionary, DLNLRR can update the dictionary in the iterative solution process, which is helpful to obtain the mapping benchmark that can better represent the subspace, and then obtain the subspace structure of data accurately. In addition, in the DLNLRR model, we can cluster the samples directly based on the LRR matrix, which can avoid the influence of spectral clustering algorithms on the clustering results.A large number of experiments in this paper show that DLNLRR can capture local structures in the data, and can quickly and accurately obtain clustering results, which has advantages in cell type recognition.

However, our method still has some limitations. More comprehensive experiments and analysis are needed. Our method has only been tested on real single cell sequencing datasets, and has not verified whether it is effective on other datasets or large-scale datasets. In addition, our method requires preset parameters, which may affect the performance of the method. The scRNA-seq data analysis still faces some challenges, such as the identification of cluster numbers, the inde-pendent selection of appropriate parameters, etc. In the next work, we will continue to explore adaptive parameter selection methods and the application of LRR on the scRNA-seq datasets, and pay attention to the development of ensemble clustering technology for scRNA-seq data analysis.

## Data Availability

There are no new data associated with this article. We downloaded Published datasets from databases provided by the National Biotechnology Information Retrieval Database (NCBI) and the European Institute for Bioinformatics (EMBL-EBI).In addition, the source code and experimental data of the DLNLRR method can be found at https://github.com/NANA-ZHANG95/DLNLRR-master.

## Abbreviations

scRNA-seq: Single-cell RNA-sequencing
SC: Spectral clustering
SSC: Sparse subspace clustering
LRR: Low-rank representation
CF: Concept Factorization
NMF: Nonnegative Matrix Factorization
NMI: Normalized Mutual Information
ARI: Adjusted Rand Index
LADMAP: the penalty term adaptive linear alternating direction
